# P-1557. Characterization of Factor H Binding Protein Expression for Neisseria Meningitidis Serogroup B Isolates, 2015–2022

**DOI:** 10.1093/ofid/ofaf695.1737

**Published:** 2026-01-11

**Authors:** Panagiotis Maniatis, Yamini Gorantla, Elise Gowen, Daya Marasini, Shalabh Sharma, Ashlesh Murthy, Kayla Weiss, Sundaram Vishwanathan

**Affiliations:** Centers for Disease Control and Prevention, Atlanta, Georgia; Centers for Disease Control and Prevention, Atlanta, Georgia; Seneca Federal Health, Chantilly, Virginia; Centers for Disease Control and Prevention, Atlanta, Georgia; Centers for Disease Control and Prevention, Atlanta, Georgia; Pfizer Inc, Pearl River, New York; Pfizer Inc, Pearl River, New York; Centers for Disease Control and Prevention, Atlanta, Georgia

## Abstract

**Background:**

Neisseria meningitidis serogroup B (NmB) is a major causative agent of invasive meningococcal disease (IMD). In the United States, the IMD NmB case rate has ranged from 0.05/100,000 to 0.01/100,000 (2015-2022). Even when treated, IMD kills 10 to 15 infected people out of 100 and, for those who survive, may lead to lifelong disabilities. Currently, there are two protein subunit licensed vaccines, MenB-fHbp and MenB-4C, both containing factor H binding protein (fHbp) as a component, the latter containing additional antigenic targets. fHbp is a critical virulence factor in complement down-regulation and fHbp surface expression levels can be correlated to human serum bactericidal (hSBA) susceptibility while evaluating the MenB-fHbp vaccine.

**Methods:**

NmB isolates collected from 2015–2022 via Active Bacterial Core surveillance and Enhanced Meningococcal Disease Surveillance were used for this study, utilizing the Pfizer-developed meningococcal antigen surface expression assay, which measures fHbp surface expression by all fHbp variants using flow cytometry. To be classified as positive, a sample must have a median fluorescence intensity (MFI) ≥ 100 and at least three times higher than that of the negative control. A sample with an MFI ≥ 1000 has been shown to correlate with a 91.2% probability of being hSBA-susceptible. A total of 574 isolates collected were tested. Based on the genomic information obtained from whole genome sequencing data using Illumina platform with data analyzed using Bacterial meningitis genome analysis platform (BMGAP), the fhbp allele present in the isolates were classified as subfamily A, subfamily B, subfamily A/B hybrid, or “not assigned.”

**Results:**

In this set, 11 isolates tested negative. Among the isolates with fHbp subfamily A or B, 77 had an MFI between 100 and 1000, while 469 showed an MFI of ≥ 1000. Two isolates with fHbp subfamily A/B hybrid and 13 isolates with FHbp not assigned had an MFI of ≥ 1000. Among the isolates containing fHbp subfamily A or B with an MFI of ≥ 1000, 61% belonged to subfamily B, while 39% were classified as subfamily A.

**Conclusion:**

Eighty-two percent of 2015–2022 isolates were above the predictive level of hSBA susceptibility, indicating strong potential coverage of these isolates by the MenB-fHbp vaccine. Funded by Pfizer.

**Disclosures:**

Panagiotis Maniatis, MS, GSK: Grant/Research Support|Pfizer: Grant/Research Support Ashlesh Murthy, MD, Pfizer Inc: Employee|Pfizer Inc: Stocks/Bonds (Public Company) Kayla Weiss, PhD, Pfizer Inc: Employee|Pfizer Inc: Stocks/Bonds (Public Company)

